# Development and Validation of a Successful Microbiological Agar Assay for Determination of Ceftriaxone Sodium in Powder for Injectable Solution

**DOI:** 10.3390/pharmaceutics4030334

**Published:** 2012-06-29

**Authors:** Patrícia V. Aléssio, Hérida R. N. Salgado

**Affiliations:** Post graduate Progam in Pharmaceutical Sciences, Faculty of Pharmaceutical Sciences Univ Estadual Paulista, Rod. Araraquara-Jaú, km 1, CEP 14801-902, Araraquara, SP, Brazil; Email: patty_alessio@yahoo.com.br

**Keywords:** ceftriaxone sodium, cephalosporins, bioassay, validation

## Abstract

Ceftriaxone sodium is a cephalosporin with broad-spectrum antimicrobial activity and belongs to the third generation of cephalosporins. Regarding the quality control of medicines, a validated microbiological assay for the determination of ceftriaxone sodium in powder for injectable solution has not been reported yet. This paper reports the development and validation of a simple, accurate and reproducible agar diffusion method to quantify ceftriaxone sodium in powder for injectable solution. The assay is based on the inhibitory effect of ceftriaxone sodium on the strain of *Bacillus subtilis* ATCC 9371 IAL 1027 used as test microorganism. The results were treated statistically by analysis of variance and were found to be linear (*r* = 0.999) in the selected range of 15.0–60.0 μg/mL, precise with a relative standard deviation (RSD) of repeatability intraday = 1.40%, accurate (100.46%) and robust with a RSD lower than 1.28%. The results demonstrated the validity of the proposed bioassay, which allows reliable ceftriaxone sodium quantitation in pharmaceutical samples and therefore can be used as a useful alternative methodology for the routine quality control of this medicine.

## 1. Introduction

The key intermediate for semisynthetic production of a large number of cephalosporins is 7-aminocephalosporanic acid, which is formed by hydrolysis of cephalosporin C produced by fermentation. Cephalosporins can be divided into first, second, third and fourth generation agents, based roughly on the time of their discovery and their antimicrobial properties [1,2,3,4,5]. 

**Figure 1:**
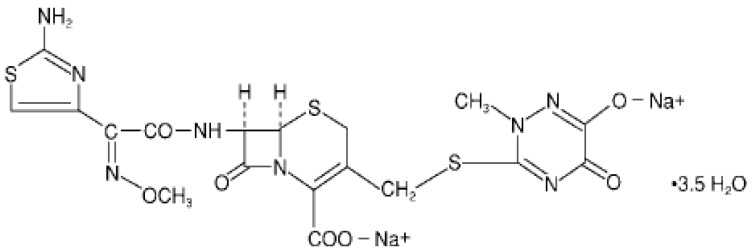
Chemical structure of ceftriaxone sodium (CAS 74578-69-1).

Among the cephalosporins, ceftriaxone sodium has excelled in the healthcare market, especially in the hospital [6]. Ceftriaxone sodium is a semisynthetic cephalosporin of the third generation with high antibacterial activity, which is widely used to treat bacterial infections caused by susceptible, usually Gram-positive organism, meningitis caused by aerobic Gram-negative bacteria and other medical conditions [6,7,8,9,10]. The chemical structure of ceftriaxone sodium is represented in Figure 1.

Ceftriaxone sodium is chemically known as, (*Z*)-7-[2-(2-aminothiazol-4-yl)-2-methoxyiminoacetylamido]-3-[(2,5-dihydro-6-hydroxy-2-methyl-5-oxo-1,2,4-triazin-3-yl)thiamethyl]-3-cephem-4-carboxylic acid, disodium salt [11,12].

Ceftriaxone sodium is administered parenterally and it penetrates into the body fluids and tissues in a satisfactory manner. Two factors contribute to the prolonged duration of action of ceftriaxone: a high fraction protein binding in the plasma and a slow urinary excretion. Ceftriaxone is excreted both in the bile and in the urine [13,14].

In the references several methods of analysis for ceftriaxone sodium were found. These methods are high performance liquid chromatography (HPLC) [15,16,17,18] fluorimetry [19,20], titration [21], spectrophotometric [21,22,23,24,25] and micellar electrokinetic capillary chromatography [26,27,28].

However, only a few methods of analysis for ceftriaxone sodium are standardized in official compendia; the literature still needs new analytical procedures aimed at speed, selectivity, low cost and simplicity of application in work routines in the quality control of pharmaceutical industries.

Hence, an attempt has been made to develop a simple, accurate and reproducible method for the determination of ceftriaxone sodium in pharmaceutical dosage forms along with method validation.

## 2. Experimental Section

### 2.1. Chemicals

The ceftriaxone sodium reference substance (assigned purity 99.8%) was supplied by Sigma-Aldrich. The lyophilized powder for injectable solutions containing 1g of ceftriaxone sodium was kindly donated by União Química Company (Pouso Alegre, MG, Brazil). All reagents used were of analytical grade. Purified water was used in all experiments.

### 2.2. Ceftriaxone Sodium Reference Solutions

An accurately weighed amount of powder equivalent to 12.5 mg of ceftriaxone sodium reference standard was transferred to 25 mL volumetric flasks and added to a phosphate buffer with pH 6.0 to obtain the final concentration of 500 μg/mL. Aliquots of 300, 600 and 1200 µL of this solution were transferred to a 10 mL volumetric flask supplemented with a phosphate buffer at pH 6.0 to obtain solutions with the concentrations of 15.0, 30.0 and 60.0 μg/mL (S1, S2 and S3), respectively that were used in the bioassay.

### 2.3. Preparation of the Sample Solutions

The content of the three vials containing an average weight of 1.1 g lyophilized powder ceftriaxone sodium was added to a closed container. A quantity of powder, equivalent to 12.5 mg of ceftriaxone sodium, was transferred to a 25 mL volumetric flask supplemented with a phosphate buffer with pH 6.0. Aliquots of this solution were transferred into flasks of 10 mL in pH 6.0 phosphate buffer solution to give concentrations of 15.0, 30.0 and 60.0 μg/mL (T1, T2 and T3), respectively, which were tested against S1, S2 and S3.

### 2.4. Microorganism and Inoculum Standardization

*Bacillus* *subtilis* ATCC 9371 IAL 1027 used in micro test showed to be more suitable due to their susceptibility to ceftriaxone sodium and the ability to form well-defined growth inhibition zones allowing accurate measurements and reduced pathogenicity compared with other microorganisms. The cultures of *B.* *subtilis* ATCC 9371 IAL 1027 were grown and maintained in culture medium Casoy (Difco, Brazil). 

The organism was standardized according to the procedure described in Brazil and the United States Pharmacopoeia [29,30]. Before use, the microorganism was cultured in Casoy broth in an Erlenmeyer flask which was incubated for 24 h at 35 ± 2 °C. Using a spectrophotometer with the wavelength set at 580 nm and an absorption cell 10 mm, the broth containing the microorganism was diluted to give a suspension containing 25 ± 2% turbidity (transmittance) using a sterile broth as a blank. From this standardized suspension, an aliquot of 1.0 mL was used in through-Grove Randal n°2.

### 2.5. Agar Diffusion Bioassay

**Figure 2:**
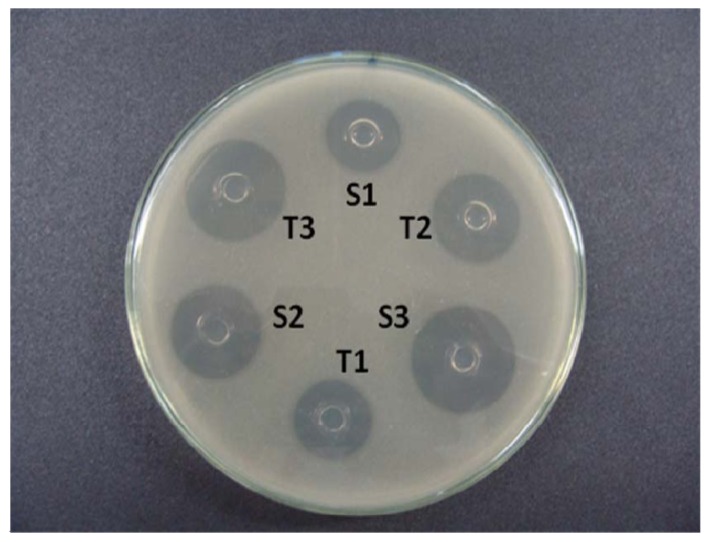
Agar diffusion assay using a strain of *B. subitilis* ATCC 9371 IAL 1027 as the test microorganism. Ceftriaxone sodium reference substance is at concentrations of 15 (S1), 30 (S2), and 60 (S3) μg/mL and the ceftriaxone sodium sample at concentrations of 15 (T1), 30 (T2), and 60 (T3) μg/mL.

The base layer of the agar medium was composed per 20 mL culture Grove Randall-1 (Difco) which was poured into a Petri dish of 100 mm × 20 mm [29,30]. After solidification of this layer, portions of 5 mL of 2-Grove Randall inoculated medium were added to 1% on the base layer. In each plate, a “template” stainless steel was distributed evenly over the surface of the inoculated medium. Alternate three holes were filled with 200 μL of the reference solutions (S1, S2 and S3), and the other three holes were filled with the sample concentration solutions (T1, T2 and T3, Figure 2). Six plates were used for each assay. The plates were incubated at 35 °C aerobically for 18 h. The diameter of the growth inhibition zone (mm) was carefully measured with a digital caliper. 

### 2.6. Calculation of Activity and Method Validation

To calculate the activity of ceftriaxone sodium, the Hewitt equation was used [31]. The assays were calculated statistically by the linear parallel model and regression analysis and verified using analysis of variance (ANOVA). The method was validated by determination of the following operational characteristics: linearity, precision, accuracy, and robustness [30,32,33,34,35,36,37,38,39,40].

#### 2.6.1. Linearity

In order to assess the validity of the assay, three doses of the reference substance were used. The linearity was evaluated by linear regression analysis, which was calculated by the least-squares method.

#### 2.6.2. Precision

The precision of the method was determined by repeatability and intermediate precision and was expressed as the relative standard deviation (RSD). The repeatability was examined by assaying six samples of ceftriaxone sodium on the same day (intraday) and under the same experimental conditions against the ceftriaxone sodium reference standard. The intermediate precision of the method was evaluated through the performance of the analysis on three days (interday) in the same laboratory.

#### 2.6.3. Accuracy

To determine the accuracy of the proposed method, the test was performed over three concentration levels, 80%, 100% and 120%, covering the specified range. Accurate aliquots of the reference standard solution (500 μg/mL) were transferred into 10 mL volumetric flasks together with aliquots of the sample solutions (500 μg/mL) and diluted with phosphate buffer solution pH 6.0 to give the final concentrations of 24.0, 30.0, and 36.0 μg/mL, (T1, T2 and T3), respectively, which were tested against S1, S2 and S3.

#### 2.6.4. Robustness

The robustness of this method was determined by analyzing the same samples under a variety of conditions. The factors considered were incubation time, incubation temperature and inoculum concentration. Analysis was performed under normal conditions and in parallel altered by assessing interference of changes in the final result.

## 3. Results and Discussion

The method was developed and validated by linearity, accuracy, precision, repeatability and robustness. For drug analysis in quality control, the simplest and fastest procedures can be applied.

The development and validation of the analytical methods for the potency determination has received considerable attention in recent years, mainly from regulatory agencies, because of their importance in pharmaceutical analysis [34,35,36,37,38,39,40].

The potency of an antibiotic may be demonstrated under suitable conditions by comparing the growth inhibition of sensitive microorganisms induced by known concentrations of the antibiotic to be examined and a reference standard [31,32].

In this experimental work of 3 × 3 design (Figure 2), three dose levels for each standard and sample, were used following the procedures described in the Brazilian Pharmacopoeia [29]. The calculation procedures normally assume a direct relationship between the observed zone diameter and the logarithm of the applied dose. The results of growth inhibition zone diameter of the ceftriaxone sodium reference substance are shown in Table 1.

**Table 1:** Diameters of inhibition zones obtained in the microbiological assay for evaluation the linearity of ceftriaxone sodium in pharmaceutical products-agar diffusion method.

	Diameter of inhibition zones (mm) *^a^*					
	P1	P2	P3	A1	A2	A3
	(15 µg/mL)	(30 µg/mL)	(60 µg/mL)	(15 µg/mL)	(30 µg/mL)	(60 µg/mL)
Day 1	16.41	18.45	20.80	16.31	18.45	20.70
Day 2	16.19	18.45	20.85	16.23	18.35	20.85
Day 3	16.20	18.30	20.75	16.01	18.45	20.90
Diameter medium	16.27	18.40	20.80	16.18	18.42	20.82
RSD% *^b^*	0.76	0.47	0.24	0.96	0.31	0.50

^a^ Mean of 3 assays with 6 plates en each; ^b^ RSD% = percentage coefficient of variation.

The representative linear equation for ceftriaxone sodium was *y* = 3.2966L*n*(*x*) + 7.26 (*n* = 3, *r* = 0.9993), where *x* is the log dose and *y* is the zone diameter. The experimental values obtained for the determination of ceftriaxone sodium in samples are presented in Table 1. These conditions must be verified by validity tests for a given probability, usually *P* = 0.05. The assays were validated by means of ANOVA, as described in the official codes. There were no deviations from parallelism and linearity with the obtained results (*P* < 0.05).

The method precision was determined by repeatability (intraday) obtaining a RSD of 1.15% and the intermediate precision was determined by analyzing the same sample on three days (between-day) with obtained RSD values of 1.40%.

The accuracy of the method was confirmed by determining the average recoveries from the samples through the method of standard addition. The mean percentage recoveries of the product were in accordance with the fixed limits of 98.0 up to 102.0, indicating the suitability of the developed method in quantifying the concentration of ceftriaxone in pharmaceutical injectable forms.

The accuracy of the method was evaluated at 80%, 100% and 120% of the nominal analytical concentration in the specified range of 15.0–60.0 μg/mL. The mean accuracy was 100.46% and RSD was 0.20% (Table 2).

**Table 2:** Values obtained in the recovery test agar diffusion method for determination of ceftriaxone sodium.

	Ceftriaxone sodium SQR added (µg/mL)	Ceftriaxone sodium SQR found *^a^* (µg/mL)	Recovery (%)	Recovery average (%)	RSD *^b^*(%)
R1	4.0	4.00	100.00	100.46	
R2	10.0	10.05	100.50		0.20
R3	16.0	16.14	100.88		

^a^ Mean of 3 assays with 6 plates en each; ^b^ RSD% = percentage coefficient of variation.

The robustness of the method was evaluated by making some modifications to the method, the results showed no significant difference demonstrating the robustness of the method, as shown in Table 3.

**Table 3:** Parameters assessing the robustness of the method microbiologically.

Variable	Range investigated	Ceftriaxone sodium *^a^* (g/vial)	Ceftriaxone sodium *^a^* (%)	RSD *^b^*(%)
Incubation time (time)	18	0.974	97.40	1.01
	24	0.992	99.23	
Incubation temperature (°C)	35	0.967	96.72	1.28
	30	0.962	96.29	
Inoculum concentration (%)	1.0	0.980	98.03	1.12
	1.2	0.996	99.68	

^a^ Mean of 3 assays with 6 plates en each; ^b^ RSD% = percentage coefficient of variation.

The quantification of antibiotic components by physicochemical methods, such as HPLC and UV spectrophotometry, although precise, cannot provide a true indication of biological activity. Therefore, bioassays continue to play an essential role in manufacturing and quality control of antibiotic medicines, and still demand considerable skill and expertise to assure success [41].

The results obtained in this study were very satisfactory, and the performed validation proved that the microbiological assay is a good alternative methodology for pharmaceutical analysis of ceftriaxone sodium lyophilized powder for injectable preparations. It is a useful analytical tool as a supplement or substitution for the physicochemical method.

## 4. Conclusions

The method using a microbiological agar assay for the determination of ceftriaxone sodium demonstrated good linearity, precision and accuracy at concentrations ranging from 15.0 to 60.0 μg/mL, therefore, being an acceptable alternative method for the routine quality control of ceftriaxone sodium in pharmaceutical forms. The method uses simple reagents with minimum sample preparation procedures and no toxically residues, encouraging its application in routine analysis.
